# Immunogenic cell death and its therapeutic or prognostic potential in high-grade glioma

**DOI:** 10.1038/s41435-021-00161-5

**Published:** 2022-01-19

**Authors:** Brecht Decraene, Yihan Yang, Frederik De Smet, Abhishek D. Garg, Patrizia Agostinis, Steven De Vleeschouwer

**Affiliations:** 1 Research Group Experimental Neurosurgery and Neuroanatomy, Department of Neurosciences, KU Leuven, Leuven, Belgium; 2 Laboratory for Precision Cancer Medicine, Translational Cell and Tissue Research Unit, Department of Imaging and Pathology, KU Leuven, Leuven, Belgium; 3 Department of Neurosurgery, University Hospitals Leuven, Leuven, Belgium; 4 Laboratory of Cell Death Research & Therapy, Department of Cellular and Molecular Medicine, KU Leuven, Leuven, Belgium; 5 VIB Center for Cancer Biology Research, Leuven, Belgium; 6 Laboratory of Cell Stress & Immunity (CSI), Department of Cellular & Molecular Medicine, KU Leuven, Leuven, Belgium; 7 Leuven Brain Institute (LBI), KU Leuven, Leuven, Belgium

**Keywords:** Immunology, Immune cell death, Neuroimmunology, Cell death and immune response, Tumour immunology

## Abstract

Immunogenic cell death (ICD) has emerged as a key component of therapy-induced anti-tumor immunity. Over the past few years, ICD was found to play a pivotal role in a wide variety of novel and existing treatment modalities. The clinical application of these techniques in cancer treatment is still in its infancy. Glioblastoma (GBM) is the most lethal primary brain tumor with a dismal prognosis despite maximal therapy. The development of new therapies in this aggressive type of tumors remains highly challenging partially due to the cold tumor immune environment. GBM could therefore benefit from ICD-based therapies stimulating the anti-tumor immune response. In what follows, we will describe the mechanisms behind ICD and the ICD-based (pre)clinical advances in anticancer therapies focusing on GBM.

## Introduction

Entering the era of immunotherapy, newly-discovered mechanisms breaking the barrier between immunity and cancer have opened the door for novel treatment paradigms. With evidence from several clinical trials, immune checkpoint inhibitors (ICI) have provided promising outcome in certain types of cancers [1–7]. However, immune checkpoint inhibitors only show their effectiveness conditionally within specific biomarker-identified subgroups of patients [8, 9]. Immunogenic cell death (ICD), discovered in the recent decades, has shed a different light on the relevance of the dialogue established between dying cancer cells and the immune system in cancer therapy [10]. ICD, named after the immunogenicity of dying/dead cancer cells, is a form of regulated cell death (RCD) induced by certain types of therapies. It is able to potentiate adaptive immune responses, targeting residual cancer cells/tissues, through the emission of endogenous molecules that acquire potent immunomodulatory functions when exposed to the extracellular environment, known as damage associated molecular patterns (DAMPs) [11]. Within the process of ICD, specifically associated to apoptotic cell death, the concomitant induction of ROS production [12] and ER stress [13] activate danger signaling, which will lead to the emission of ICD-associated DAMPs in a spatio-temporal manner [14, 15]. Altogether the release of these immunomodulatory molecules, by binding to cognate pattern recognition receptors on the surface of antigen-presenting cells will function as adjuvants to promote their chemotaxis and maturation, which together with the uptake of tumor antigens from dying/dead cells, will culminate in the activation of adaptive immune responses. The ability to activate danger signaling pathways unleashing the proinflammatory/immunomodulatory potential of DAMPs, is therefore considered the dominant process distinguishing ICD from tolerogenic cell death [16].

ICD is rapidly gaining popularity in the field of anti-cancer therapy. Some conventional treatments have shown to be able to induce some form of ICD [17], and currently, new ICD inducers are under screening [18, 19]. Besides, some ICD inducers can function in synergy with other types of immunotherapy, such as immune checkpoint inhibitors therapy [20] to enhance their effectiveness.

### Main molecular and immunological features of ICD

To induce cell death with increased immunogenicity, an ICD inducer is necessary in the first place [19–27]. Known therapeutic treatments associated with ICD comprise a variety of cellular stressors, including (but not limited to) conventional chemotherapies (e.g. various anthracyclines), proteasomal inhibitors, oncolytic viruses, and physicochemical/physical stressors such as radiotherapy, photodynamic therapy (PDT), high-hydrostatic pressure [28]. However, with the screening of conventional anti-cancer therapy and the discovery of novel medications for their potential ability to induce ICD, the collection of drugs or treatments associated with a stress-induced RCD with inflammatory and immunogenic features, continues to increase. Based on the molecular knowledge of the signaling pathway triggered by drugs or treatments eliciting cellular stress-associated to ICD, ICD inducers can be classified into two main categories, designated as type I and type II. Type I ICD inducers are typified by genotoxic drugs, like anthracyclines, causing the activation of the unfolded protein response and reactive oxygen species production [12] as secondary or collateral cytoplasmic processes evoked in response to damage to their main intracellular target (i.e. DNA). Different from these agents, type II inducers, such as hypericin-mediated PDT, cause ER-focused reactive oxygen species formation, thereby prompting loss of ER homeostasis, intracellular Ca^2+^ elevation and fast danger signaling pathways eliciting the exposure and release of DAMPs [29–31]. As a result, type II ICD inducers are usually more robust than type I inducers in terms of ICD propensity.

The ER chaperone calreticulin (CRT), is usually translocated from the ER to the plasma membrane during the early phase of ICD as the response of ER stress. The mobilization of CRT to the PM during the early phase of apoptosis has been shown to require loss of the ER-Ca+ store, BAX/BAK, the recruitment of caspase-8, and PERK which partaking the process of unfolded protein response [14]. Depending on the type of ICD, either eIF2a the downstream effector of PERK or likely PERK-scaffolding function, has been found to be crucial for CRT trafficking to the plasma membrane [32]. Once exposed at the surface CRT acts as potent ‘eat-me’’ signal, by binding to CRT cognate receptor on antigen-presenting cells, and facilitates the engulfment of dying cells by DCs. Clinical studies have shown that, in human non-small cell lung cancers, the expression level of CRT is also positively correlated with accumulation of matured DCs and the survival of patients [33]. In neuroblastoma, expression of CRT can be used as an independent prognostic factor [34], suggesting the biomarker potential of CRT.

Adenosine triphosphate (ATP), is secreted during a pre-mortem phase of ICD. During ICD, ATP secreted by stressed cells, by binding to purinergic receptors (P2RY2 and P2RX7) on the surface of DCs, acts as ‘find-me’’ signal to recruit DCs to the site of dying cancer cells and stimulates the assembly and activation of the inflammasome, leading to the production and secretion of interleukin (IL)-1β [35] by DCs. The pathway causing ATP release from stressed cancer cells is, however, dependent on the type of ICD inducers. Autophagy has been shown to be either required for ATP release in response to anthracyclines, or be dispensable in case of Hypericin-PDT [31]. In the last settings, autophagy induction after Hypericin-PDT by eliminating oxidatively damaged proteins, attenuated ER stress, and the exposure of CRT [31]. In contrast, Prieto et al. showed that in response to P2Et extract from the plant *Caesalpinia spinosa*, autophagy occurs before apoptotic cellular demise to promote ecto-CRT [36] and further immune response will be elicited during the progression of ICD [31]. Hence the role of autophagy in ICD remains highly context-dependent.

In the post-demise phase of ICD, nuclear HMGB1 is relocated to the cytoplasm and will be exposed extracellularly upon plasma membrane rupture. This passive released HMGB1, during the later stage of apoptosis and secondary necrosis, stimulates tumor antigen presentation DC maturation by binding toll-like receptor 4.

Besides these DAMPs which have an intracellular housekeeping function, other danger molecules can be transcriptionally induced during ICD. For example, certain type of ICD inducers, triggers type I interferon (IFN) responses and the secretion of several chemokines [37] with a pronounced immunomodulatory role. It is thought that stimulation of type I IFN activity is one of the reasons why ICD can function synergistic with anti-PD1 therapy [38]. For example, radiotherapy induction of type I IFN can overcome the resistance of anti-PD1 [39]. On the other hand, since persistent type I IFN response will lead to immunosuppression only ICD inducers that cause transient type I IFN release may possess the beneficial effect of stimulating anti-tumor immunity. In line with this, dinaciclib, a cyclin-dependent kinases inhibitor that can induce a proper amount of type I IFN release in a timely manner, alleviates the resistance to checkpoint-blockade treatment [20, 40]. Intriguingly, a recent study shows that transcriptional pro-inflammatory signature is shared by both anthracyclines and Hypericin-PDT, and distinguishes ICD from non-ICD (e.g. Cisplatin) regimens. This ICD-associated response, which is driven by the activation of NF-kB and AP-1 transcritption factors coordinated by heat shock protein 60 [37] is critical for the anticancer vaccination potential of ICD-inducing chemotherapy.

However, it should be noticed that both ICD-associated DAMPs and various cytokines and chemokines induced by ICD can have profound and sometimes contrasting impact on the TME. Surface exposed calreticulin for example promotes tumor antigen presentation by facilitating DC phagocytosis, but can also promote cancer cell invasion [41]. HGMB1 stimulates DC maturation, however, its immunomodulatory activity is dependent on its oxidation status [42] and its role is tumor and TME specific [43]. Extracellular ATP acts as a chemoattractant for immature DCs but can be converted into an immunosuppressive form (adenosine) by CD39 (ecto-nucleoside triphosphate diphosphohydrolase 1) and CD73 (ecto-5’-nucleotidase). CD39 performs the first step converting ATP into AMP and CD73 further converts it into adenosine. Furthermore, both enzymes regulate the magnitude of the purinergic reaction surrounding the immune cells. High expression of CD73 is therefore associated with low levels of lymphocytes in the TME and poor prognosis in for example colorectal, prostate, and triple-negative breast cancer [44–47]. Of note, CD73 is regulated by HIF-1alpha and therefore more abundant in a hypoxic microenvironment [48]. Finally, ICD may exert several effects on the TME [49]. Phagocytosis of ICD-dying tumor cells by DC will elicit full DC maturation, and release of immunogenic cytokines (e.g., IL-6, IL-2,…), which in turn will promote differentiation and proliferation of CD4+ and CD8+ T lymphocytes and thus ameliorate the adaptive immune response. Tumoral DCs are therefore associated with a more favorable prognosis [33]. Apart from that, as mentioned above, various anticancer regimens may cause the secretion of type I IFN by dying cancer cells which will further favor T lymphocyte recruitment and the establishment of a strong adaptive anticancer immunity. A pleiotropy of antitumor effects is kickstarted by those T-cells ranging from stimulatory feedback loops to IFN release (which has an anti-angiogenic, anti-proliferative, and pro-apoptotic effect) and from complex interactions between subtypes of mainly T lymphocytes, resulting in antitumor attacks, to chemokine expression (CXCR3) which attracts other immune cells towards the tumor. Also the latter effect may be indirectly caused by IFN [50]. Furthermore, a neutrophilic inflammation reaction is also seen in ICD [51].

### Immunogenicity of different types of RCDs

Before the discovery of ICD, apoptosis was generally interpreted as non-immunogenic regulated cell death manner (Table 1). However, in autoimmune diseases, apoptotic cellular antigen has long been identified as a target of autoantibodies in autoimmune diseases, which hints the relation between apoptosis and immunity [52, 53]. Early study showed that under certain subclass of therapies, apoptosis induced can be immunogenic and pro-inflammatory [54]. Subsequent studies have demonstrated that using various ICD inducers, apoptotic cancer cells can be used as cancer vaccines causing tumor regression to different extents in different cancers. However, as mentioned above, other types of RCD typically associated to more robust inflammatory responses, have been shown to elicit ICD.

**Table 1:** Studies on immunogenicity/ICD potential of different types of RCDs.

Study	ICD inducer	PCD	DAMPs	Other proof of immunogenicity
Casares et al. (2005) [27]	Doxorubicin (DX)	Apoptosis	HMGB1, HSP70	
Tesniere et al. (2010) [25]	Oxaliplatin (OXP)	Apoptosis	Ecto-CRT, HMGB1	
Panzarini et al. (2014) [26]	Rose Bengal Acetate Photodynamic Therapy (RBAc-PDT)	Apoptosis	Ecto-CRT, ATP, HMGB1, HSP70, and HSP90	
Koks et al. (2015) [136]	Newcastle disease virus (NDV)	Necroptosis	Ecto-CRT, ATP, HMGB1, HSP70, and HSP90	
Aaes et al. (2016) [56]	Doxycycline (doxy)/doxy + B/B dimerizer	Necroptosis	ATP and HMGB1	DC maturation
Teo et al. (2017) [140]	BYL719 (PI3Ka inhibitor) + LEE011(CDK4/6 inhibitor)	Apoptosis	Ecto-CRT	
Hossain et al. (2018) [20]	Dinaciclib	Apoptosis	Ecto-CRT, ATP, and HMGB1	
Li et al. (2018) [137]	Doxorubicin-polyglycerol-nanodiamond composites	Apoptosis	Ecto-CRT, HMGB1, and HSP90	DC maturation
Turubanova et al. (2019) [21]	Photosens	Apoptosis and ferroptosis	Ecto-CRT, ATP, and HMGB1	IL-6
Turubanova et al. (2019) [21]	Photodithazine (PD)	Apoptosis	Ecto-CRT, ATP, and HMGB1	IL-6
Efimova et al. (2020) [62]	RAS-selective lethal 3	(Early) ferroptosis	ATP and HMGB1	DC maturation
Franco-Molina et al. (2020) [141]	Panobinostat (PAN)	Apoptosis	HMGB1, HSP70, and HSP90	
Franco-Molina et al. (2020) [141]	*Lophophora williamsii (LW)*	Apoptosis	HMGB1, HSP70, and HSP90	
Jeong et al. (2021) [23]	Fluorinated mitochondria-disrupting helical polypeptides (MDHPs)	Apoptosis	Ecto-CRT, ATP, and HMGB1	
Villamañan et al. (2021) [142]	Temozolomide (TMZ) + CX-4945 (protein kinase CK2 inhibitor)	Unspecified	Ecto-CRT and ATP	
Turubanova et al. (2021) [22]	Porphyrazines (pz I)-PDT	Apoptosis	ATP and HMGB1	DC maturation
Turubanova et al. (2021) [22]	Porphyrazines (pz III)-PDT	Apoptosis and Necroptosis	ATP and HMGB1	DC maturation
Tomić et al. (2021) [24]	Plasma-activated medium (PAM)	Apoptosis		DC maturation

Necroptosis is ‘programmed’’ by the activation/phosphorylation of receptor-interacting protein kinase-1 (RIPK-1), RIPK-3, and mixed lineage kinase domain-like pseudokinase regulated pathway, ultimately causing the permeabilization of plasma membrane [55], Necroptosis is an alternative RCD that could elicit ICD [56] especially in apoptosis-resistant cancer cells subpopulations [57–59]. Under certain circumstances, necroptotic cancer cells can induce ATP secretion and CXCL1 release, become phagocytized by DCs and stimulate their maturation. To be noted, in the study of Aaes et al. [60], necroptotic cancer cells failed to cause ER stress and the translocation of CRT from ER to plasma membrane. Further in vivo study validated that necroptotic cancer cells can induce a potent immune response by the cross-priming, proliferation, and cytokine release of cytotoxic T-cells [56]. Moreover, necroptosis has been shown to result in a higher presence of CD8+ T-cells and to reduce the number of myeloid-derived suppressor cells in pancreatic tumors [61].

Over the last decade more types of RCD have been defined and their immunogenicity was subsequently studied. Ferroptosis, an iron-mediated and lipid peroxidation-driven necrotic cell death, can induce the secretion of ATP and the release of HMGB1 in fibrosarcoma and glioma [62]. However, the immunomodulatory function of ferroptosis may depend on the cell death stage. A recent study shows that, only in the early stage, ferroptosis can promote the maturation of DCs, but not in the late stage [62]. Hence whether, when, and how ferroptosis might open another door of prompting ICD, especially when other RCDs are ‘silenced’’ in cancer cells is still unclear. More in-depth studies are needed to further understand the mechanistic underpinnings of this form of RCD and its impact on immune responses.

Besides, different types of RCD can exist simultaneously in ICD induced by a single inducer. Turubanova et al. showed that in photosens -PDT induced ICD, cell death can be inhibited by both zVAD-fmk (apoptosis inhibitor) and ferrostatin-1 and DFO (ferroptosis inhibitors), which means apoptosis and ferroptosis are co-existing during the process [22]. Further research on the cross-talk of different RCDs is warranted [63]. Moreover, the anti-tumor immunity potential of other genetically defined necrotic cell death processes, like secondary necrosis, pyroptosis, and PAN-optosis, which integrates pyroptosis, apoptosis, and necroptosis into a unified programmed cell death behavior, is currently under investigation [64].

### Cell autonomous mechanisms of ICD evasion

A hallmark of cancer cells is evasion from the surveillance of the immune system [65]. While various cancer cell autonomous and non-autonomous factors contribute to this complexity and are still under investigation, it is intriguing that tumor cells may evade ICD by the chronic deregulation of processes regulating cell proteostasis, such as the unfolded protein response and autophagy, that contributes to the immunogenicity of the stressed/dying cancer cells. Clearly, acute activation of the lethal arm of ER stress by therapy-induced cellular stress and death pathway, harness the ‘danger’’ component of this stress response, in a fashion similar to that induced by microbial pathogens, thus turning sterile cancer cell death into a mimicry of pathogen-induced cell demise, with consequent activation of immune responses.

However, certain cancer cell autonomous mechanisms regulating proteostasis can either subvert danger signaling pathways (like the PERK-eiF2α axis [66]), cause retention of DAMPs thereby impairing the proficient dialogue between dying cancer cells and the immune system or secrete mutate forms of DAMPs (like mutant CRT) which supposed to act as a decoy for DCs in the wild-type [67]. For example, in glioma Bip upregulation, a typical marker of the activation of the unfolded protein response, restricted DAMPs exposure and release in glioma stem cells [68]. A recent study also reported that cancer cells may avoid the exposure of CRT through a mechanism involving stanniocalcin-1 mediated retention of CRT in the cytoplasm (thus suggesting a pool of cytosolic CRT), a process that impairs phagocytosis by antigen-presenting cells and subsequent anticancer adaptive immunity [67]. Hence, strategies designed to target deranged proteostasis in cancer cells in order to reinstate the cancer cell-immune cell dialogue, will require an increased knowledge of the inhibitory elements of the danger signaling pathway elicited by ICD.

### ICD and Glioblastoma

**Fig. 1:**
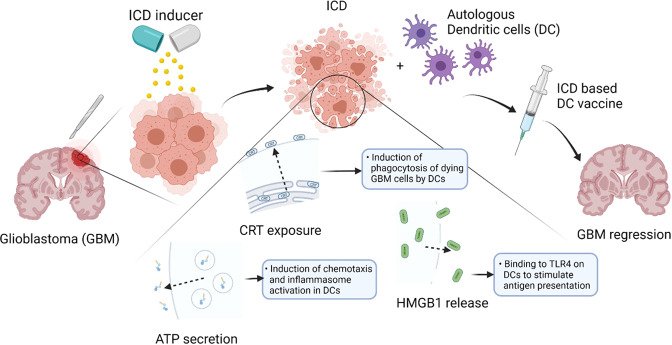
Clinical setting of ICD-based GBM vaccination. After GBM resection, ICD will be induced using GBM cells from resected tumor tissue. Next, DC vaccine are prepared ex vivo by exposing autologous DCs to GBM cells dying through ICD. The vaccine contains GBM cells, which are avitalized after ICD induction, and DAMPs, which are either exposed, secreted or released. After applying to the patient, with tumor antigen presented by DCs, effective and GBM-specific T-cell response will be triggered and augmented. Remaining GBM cells will then be targeted to suppress GBM growth and regression. As a result, prolonged survival of the patient might occur.

Glioblastoma, a grade IV glioma, is the most aggressive type of primary brain tumor with a dismal prognosis of approximately 15 months under standard of care therapy which consists of maximal safe surgical resection followed by both radiotherapy and chemotherapy (Temozolomide) [69, 70]. However, a minority of patients, estimated to be around 3% of all GBM patients, can live up to five years or longer [71]. This contributes to the new vision on GBM where it is not considered to be one fixed entity but rather an inter- and intrapersonal heterogeneous tumor behaving differently among patients. Several immunological reasons for the poor prognosis in GBM have been postulated. Amongst these the most important ones seem the GBM-associated lymphopenia, the ‘cold state’’ of these tumors depriving them from effector T-cell infiltration, their inability to become fully activated, and the formerly mentioned heterogeneity [72]. Over the past years there has been increasing evidence pointing towards the propensity to undergo ICD as a prognostic factor linked with longer survival in cancer patients in general including GBM patients [3]. Fitting in the ‘heterogeneity picture’’ there is the observation that a tumor with a higher ICD propensity could elicit a stronger anti-tumor immune response and as such could combat and slow down tumor growth more efficiently [73]. Subsequently, this would also result in a stronger anti-tumor ‘self-vaccine’’ response (Fig. 1).

The GBM tumor microenvironment consists of tumor cells (from glioma stem cells to fully differentiated tumor cells), resident (microglia), and infiltrating immune cells (in GBM mainly macrophages and much less T-lymphocytes), structural stromal (endothelial, astrocytes, oligodendrocytes), and neuronal cells. Whereas the direct, innate immune response (which consists of cells like macrophages, a few NK cells, and others) is the first line of defense against tumor cells, it also primes a more precise and stronger response called ‘the adaptive immune response’ (of which lymphocytes are the main cell type). This response recognizes tumor antigens presented by the proper major histocompatibility complex (MHC) molecules. Although in broad terms of e.g. T-cell infiltration, the immune framework in most GBMs looks similar, the exact immune cell composition of the tumor microenvironment varies over time (for example primary versus recurrent GBMs) and space (‘‘intratumoral heterogeneity’’) [74]. The latter indirectly implies the complexity of all possible interactions that can take place between immune and/or tumor cells within the tumor microenvironment. Furthermore, the constitution can also be modified by external factors, like chemo-or radiotherapy [75, 76].

Certain therapies used in the treatment of brain tumors, can induce the main cell death-associated hallmarks of ICD [27, 77]. pushing the final effect on the tumor beyond the results of the initiating therapeutic mechanisms of e.g chemo-and radiotherapy. [4] However, how GBM heterogeneity and the associated tumor microenvironment enact cancer cell-intrinsic resistance mechanisms blunting the responses to potential ICD-inducing regimens remains incompletely understood.

### ICD-inducing modalities in GBM

Several treatment modalities can induce ICD and thus an anticancer immune response reinforcing the effects of conventional treatment methods (Table 2). However, so far only a few bona fide ICD inducers have been identified and were tested in clinical trials [78, 79].

**Table 2:** Studies supporting the clinical evidence behind ICD-based effects of treatment modalities in GBM.

Main ICD-based treatment modality	Clinical evidence in GBM	Oxford level of evidence	Number of patients receiving the therapy	Articles supporting this evidence (ref)	ICD/immune response measured?*
5-aminolevulinic acid (PDT)	RCT	1b	13	Ejmael et al. (2007) [143]	No
	Cohort study	2b	10	Beck et al. (2007) [144]	No
	Cohort study	2b	5	Johansson et al. (2013) [145]	No
	‘‘Outcomes’’ research	2c	15	Schwartz et al. (2015) [146]	No
Radiotherapy	Only preclinical studies available [95, 96]				
Chemotherapy Temozolomide (in combination with a peptide-based vaccine)	RCT	1b	22	Sampson et al. (2010) [147]	No
Tumor treating fields	RCT	1b	120	Stupp et al. (2012) [123]	No
	‘Outcomes’ research	2c	457	Mrugala et al. (2014) [148]	No
	RCT	1b	466	Stupp et al. (2017) [124]	No
	RCT	1b	475	TRIDENT (ongoing)	No
DC-vaccination	Cohort study	2b	7	Yu et al. 2001 [149]	T-cell infiltration
	Cohort study	2b	7	Yamanaka et al. (2003) [150]	T-cell infiltration
	Cohort study	2b	9	Yu et al. (2004) [151]	T-cell infiltration
	Cohort study	2b	12	Liau et al. (2005) [152]	TGF-β_2_ expression
	Cohort study	2b	56	De Vleeschouwer et al. (2008) [153]	Immune response
	Cohort study	2b	32	Wheeler et al. (2008) [154]	IFN & immune response
	Cohort study	2b	10	Chang et al. (2011) [155]	T-cell infiltration
	Cohort study	2b	16	Fadul et al. (2011) [156]	IFN & T-cell response
	Cohort study	2b	77	Ardon et al. (2012) [157]	Immune response
	Cohort study	2b	18	Cho et al. (2012) [158]	No
	Cohort study	2b	7	Akiyama et al. (2012) [159]	Immune response
	Cohort study	2b	13	Jie et al. (2012) [160]	Immune response
	Cohort study	2b	20	Shah et al. (2013) [161]	TGF-β_2_ expression
	Cohort study	2b	21	Phuphanich et al. (2013) [162]	Tumor-associated antigens; immune response
	Cohort study	2b	7	Vik-Mo et al. (2013) [163]	Immune response
	Cohort study	2b	6	Sakai et al. (2015) [164]	T-cell response
	Cohort study	2b	14	Hunn et al. (2015) [165]	IFN & immune response
	Cohort study	2b	11	Akasaki et al. (2016) [166]	Immune response
	Cohort study	2b	22	Batich et al. (2017) [167]	IFN & T-cell response
	RCT	1b	32	Inognés et al. (2017) [168]	IFN & immune response
	RCT	1b	232	Liau et al. (2018) [169]	No
	RCT	1b	22	Yao et al. (2018) [170]	IFN & immune response
	RCT	1b	17	Reap et al. (2018) [171]	IFN & immune response
	RCT	1b	81	Wen et al. (2019) [172]	IFN & immune response
	Cohort study	2b	5	Wang et al. (2020) [173]	IFN & immune response
	RCT	1b	15	Mitsuya et al. (2020) [174]	IFN & immune response

*While some studies measured immune (macrophages, T-cells,…) - or ICD-associated features (DAMPS) others only described the clinical effect (outcome, quality of life,…) of ICD-inducing therapies.

Photodynamic therapy (PDT) has been tested in several cancers, among which GBM [80]. One large review of literature looking at over 1000 patients in several observational studies concluded PDT to be a safe and beneficial treatment method leading to a significant prolongation of good quality survival [81]. However, the quality of the included studies is limited as no randomized controlled trials are performed yet. Moreover, it is worth mentioning that PDT is less effective in the hypoxic niche, as well that it itself exacerbates hypoxia [82, 83]. Currently, different strategies are being developed to tackle this issue [84–86]. It is to notice that one of the main forms of PDT is 5-aminolevulinic acid which is a prodrug metabolized in high grade glioma into a fluorescent metabolite and commonly used to guide maximal safe resection in newly diagnosed and recurrent GBM [87].

Radiotherapy was also found to possess ICD inducing properties in several cancers [88]. It can render the tumor microenvironment more immunogenic by increasing MHC and cell death receptor expression thereby improving the killing of irradiated tumor cells by NK cells and T-cells [89]. It further expresses chemokines (CXCL16) and increases IFN -γ production contributing to T-cell infiltration and recognition of tumor cells by CD8+ T-cells [90–92]. Up till now, a biomarker to predict the ICD inducing capacity of Radiotherapy in cancer has not been identified [93]. Radiotherapy as a standalone ICD inducer is rarely studied in clinical trials and the potential benefit is more often explored in combinational treatment methods. E.g., in breast cancer, the combination of radiotherapy and Imiquimod (a topical TLR7 agonist) decreases recurrence rates and improves primary tumor response [94]. By analyzing the concentration of HMGB1 and of Hsp70 in supernatants of glioblastoma cell cultures treated with fractionated Radiotherapy an increase in these DAMPs was seen [95, 96] (In this studies the ICD inducing capacity of temozolomide was found to be limited). Up till now, no clinical trials have been performed in glioma.

Several chemotherapeutic agents have shown to induce ICD; paclitaxel, anthracyclines, and anthracycline-derivatives with bleomycin and doxorubicin being the most known ones [27, 97, 98]. In glioma mouse models cyclophosphamide was found to initiate ICD, but evidence in glioma patients is lacking [99]. Furthermore, prolonged oxaliplatinum treatment resulted in elevated translocation of calreticulin to the cell surface in glioma cells [100]. It is also worth mentioning that not all chemotherapeutic agents induce ICD, although the exact mechanism why some do and others don’t is still under investigation. Furthermore, an extrapolation of the ICD inducing ability of chemotherapeutic agents in extracranial cancers to brain tumors is not correct. While for example platinum compounds like cisplatinum induce ICD in several extracranial cancers, this effect was not seen in primary brain tumors [25, 101]. Studies investigating the effect of ICD-inducing chemotherapeutic Oxaliplatin in combination with oncolytic viruses in colorectal cancer in mice saw an additional effect leading to diminished tumor growth and longer median survival [102]. This is in line with earlier studies showing the ICD inducing capacity of oncolytic viruses [103, 104].

Chemotherapeutic drugs loaded in a tumor microenvironment -responsive nanoparticle and injected into a tumor improved the ICD effect, released more DAMPs, or increased immune infiltration of DCs and T lymphocytes compared to free delivered drugs [105]. In PTEN-negative orthotopic GBM epirubicin-loaded micelles in combination with anti-PD1 therapy overcame the weakening of antitumor effects of immune checkpoint inhibitors normally caused by lymphodepletion when administered systemically [106]. Another nanotechnique used is called ‘nanopulse stimulation’’. Here a very short electrical pulse is repeatedly administered at a high amplitude to the tumor, subsequently triggers ER stress, and therefore acts as an ICD-inducer [107]. However, both nanotechniques are still in their infancy.

Near-infrared photoimmunotherapy is another recently developed technique. This hybrid technique consists of an antibody that targets specific tumor antigens and a photo-activating, phthalocyanine-based chemical, IRDye700DX that attracts the NIR light. This light triggers cytotoxic reactions in the targeted cancer cells causing ICD [108]. Increased levels of DAMPs were seen when near-infrared photoimmunotherapy was applied implying its ICD inducing effect. Further clinical trials are on their way.

Other less known ICD inducers are high hydrostatic pressure and hyperthermia. High hydrostatic pressure induces the expression on the cell surface and the release of DAMPs on a wide variety of human tumor cells (leukemia, ovarian cancer, and prostate cancer) [109]. Hyperthermia (ranging from 41 °C to 44 °C) includes local as well as whole body administration of heat. It has several effects on the tumor microenvironment including improvement of antigen presentation, maturation, and migration of DCs, and also facilitates migration of T-cells to lymph nodes. The main disadvantage is collateral damage of the heat to non-tumoral regions, although the nanoparticle carrier technique discussed above is being explored here as well. The clinical significance of this technique is currently limited to combination treatments with chemotherapy or immunotherapy and the radiosensitization effect of hyperthermia. Studies were done in breast, gastrointestinal, cervical, and head-&-neck cancer [110–114]. In the brain, induction of hyperthermia is being hampered by obvious safety constraints but with more accurate thermal monitoring tools, controlled hyperthermia is being explored in brain tumors as well. Examples are laser interstitial thermal therapy and high-intensity focused ultrasound. Laser interstitial thermal therapy is a percutaneous ablative procedure in which thermal laser energy is delivered via an optic fiber probe precisely into the tumor under stereotactic guidance [115]. It is being used in several brain tumors, especially in non-resectable GBM, although large clinical trials concerning the exact benefit and application are still lacking. High-intensity focused ultrasound is another non-invasive intracranial ablation technique in which a focused beam of ultrasound rays is targeted at a limited tumoral region to maximize local energy accumulation causing tumoral tissue destruction [116]. Several small case studies have been published showing a survival advantage using this technique in GBM patients [117–119]. However Medel et al.,61 postulated that GBM might not be the ideal pathology for this treatment modality and it might be more successful for well-circumscribed tumors, such as metastases or low-grade brain tumors, where surgery cannot be performed [120, 121].

Also, it was discovered that certain targeted drugs, epidermal growth factor receptor inhibitors and tyrosine kinase inhibitor Crizotinib, might also exert an ICD inducing effect although both have only been tested in mice [18, 122].

Specifically, for GBM, ‘Tumor treating fields’’ is a novel clinically integrated treatment modality with ICD potential [123–125]. This technique, first described in 2004, uses very-low-intensity, intermediate-frequency alternating electrical fields that exert several antitumoral effects. The induction of ICD is one of the key mechanisms behind this therapy, next to neovascularization, antimitotic activity, and inhibition of cancer cell migration, invasion, and proliferation [125]. It was proven to be both effective and safe in GBM-patients in two-phase three RCTs [123, 124]. Although tumor treating fields showed an increase in overall survival in both newly diagnosed and recurrent GBM, the preferable combination with other currently used anticancer modalities should be further investigated [126].

Another promising technique is DC-based vaccination (Fig. 1). In a high-grade glioma mouse model harnessing the potential of Hypericin-PDT based DC vaccines, these vaccines reduced the immunosuppressive GBM burden and synergized with the anti-GBM action of temozolomide and resulted in an increased overall mice survival of approximately 300% [127] Interestingly, the efficacy of stressed/dying cells after Hypericin-PDT to induce DC maturation and the overall efficiency of DC vaccines, were abolished by the neutralization of the main ICD-associated DAMPs namely HMGB1, ATP and CRT [128]. This is in line with the finding that, in contrast to anthracyclines or other regimens, Hyp-PDT mediated ICD is not associated to the stimulation of Type I IFN responses [16, 129, 130]. Considering that in about 50% GBM patients, type I and/or type II IFN family genes are deleted intrinsically [131], this suggests that Hypericin-PDT elicited immunogenicity will not be compromised and should be considered for its potential clinical application in GBM.

Several, mostly small, clinical trials have been published (Table 1). In general, they point towards a small benefit in terms of survival in combination with other treatment modalities. However, substantial and significant improvements were not yet found. The heterogeneity in-between GBM tumors may be a possible underlying explanation in the discrepancy seen in vaccine responses.

Another recent experimental technique described in a GBM mouse models is an injectable hydrogel system that can be delivered into the postsurgical tumor cavity. It subsequently induces ICD and results in a sustained T-cell infiltration, therefore mimicking a hot tumor immune environment which combats local tumor remnants, preventing recurrence. Both a prolonged survival and decreased tumor relapse were seen [132].

Other less known ICD-based techniques that are currently being developed are genetically engineered viruses (oncolytic viruses), which uses viral vectors (and thus unable to replicate) to deliver cytotoxic material to the tumor cells resulting in ICD [133–135]. Also naturally occurring oncolytic viruses have been described [136]. Another modality are protein kinase CK2 inhibitors, which shows already at low concentrations cytotoxic activity in GL261 GB cells, inducing ICD; DC-mediated delivery of doxorubicin-polyglycerol-nanodiamond composites, a potent DAMPs inducer [137]; as well as liposomes modified to cross the blood-brain barrier and loaded with the chemotherapeutic drug Doxorubicin [138].

Finally, although necroptotic components were found in GBM, the influence on the immune environment was to our knowledge never examined [139].

## Conclusion

ICD is rapidly gaining research momentum as a key-mechanism to pursue in effective and sustainable cancer therapies. Current evidence of its importance in glioma therapies is often indirect, scattered and inconclusive but in analogy with many other tumor types, ICD propensity could become a pivotal prognosticator for long-term disease control and continues to capitalize on its -at least theoretical -potential for cure.
