# Publisher Correction: Immunogenic cell death and its therapeutic or prognostic potential in high-grade glioma

**DOI:** 10.1038/s41435-022-00187-3

**Published:** 2022-11-04

**Authors:** Brecht Decraene, Yihan Yang, Frederik De Smet, Abhishek D. Garg, Patrizia Agostinis, Steven De Vleeschouwer

**Affiliations:** 1 Research Group Experimental Neurosurgery and Neuroanatomy, Department of Neurosciences, KU Leuven, Leuven, Belgium; 2 Laboratory for Precision Cancer Medicine, Translational Cell and Tissue Research Unit, Department of Imaging and Pathology, KU Leuven, Leuven, Belgium; 3 Department of Neurosurgery, University Hospitals Leuven, Leuven, Belgium; 4 Laboratory of Cell Death Research & Therapy, Department of Cellular and Molecular Medicine, KU Leuven, Leuven, Belgium; 5 VIB Center for Cancer Biology Research, Leuven, Belgium; 6 Laboratory of Cell Stress & Immunity (CSI), Department of Cellular & Molecular Medicine, KU Leuven, Leuven, Belgium; 7 Leuven Brain Institute (LBI), KU Leuven, Leuven, Belgium

**Keywords:** Immunology, Immune cell death, Neuroimmunology, Cell death and immune response, Tumour immunology

Correction to: *Genes & Immunity* 10.1038/s41435-021-00161-5, published online 19 January 2022

## Abstract

Immunogenic cell death (ICD) has emerged as a key component of therapy-induced anti-tumor immunity. Over the past few years, ICD was found to play a pivotal role in a wide variety of novel and existing treatment modalities. The clinical application of these techniques in cancer treatment is still in its infancy. Glioblastoma (GBM) is the most lethal primary brain tumor with a dismal prognosis despite maximal therapy. The development of new therapies in this aggressive type of tumors remains highly challenging partially due to the cold tumor immune environment. GBM could therefore benefit from ICD-based therapies stimulating the anti-tumor immune response. In what follows, we will describe the mechanisms behind ICD and the ICD-based (pre)clinical advances in anticancer therapies focusing on GBM.

This article is part of the Special Issue “Immunology of cell death in cancer & infection”, Guest Editor: Professor Abhishek D. Garg, Katholieke Universiteit Leuven, Belgium. It was unintentionally published in issue 23, 1–11 (2022). You can access the article via this link: https://www.nature.com/articles/s41435-021-00161-5. We apologise for the inconvenience.

